# Quantification of microcirculatory blood flow: a sensitive and clinically relevant prognostic marker in murine models of sepsis

**DOI:** 10.1152/japplphysiol.00793.2014

**Published:** 2014-12-04

**Authors:** Claire A. Sand, Anna Starr, Catherine D. E. Wilder, Olena Rudyk, Domenico Spina, Christoph Thiemermann, David F. Treacher, Manasi Nandi

**Affiliations:** ^1^British Heart Foundation Centre for Cardiovascular Research, King's College London, London, United Kingdom;; ^2^Pharmacology and Therapeutics, Institute of Pharmaceutical Science, King's College London, London, United Kingdom;; ^3^The William Harvey Research Institute, Barts and The London School of Medicine & Dentistry, Queen Mary University of London, London, United Kingdom; and; ^4^Department of Intensive Care, Guy's & St. Thomas NHS Foundation Trust, London, United Kingdom

**Keywords:** animal models of cardiovascular disease, microcirculation, sepsis, septic shock

## Abstract

Sepsis and sepsis-associated multiorgan failure represent the major cause of mortality in intensive care units worldwide. Cardiovascular dysfunction, a key component of sepsis pathogenesis, has received much research interest, although research translatability remains severely limited. There is a critical need for more comprehensive preclinical sepsis models, with more clinically relevant end points, such as microvascular perfusion. The purpose of this study was to compare microcirculatory blood flow measurements, using a novel application of laser speckle contrast imaging technology, with more traditional hemodynamic end points, as part of a multiparameter monitoring system in preclinical models of sepsis. Our aim, in measuring mesenteric blood flow, was to increase the prognostic sensitivity of preclinical studies. In two commonly used sepsis models (cecal ligation and puncture, and lipopolysaccharide), we demonstrate that blood pressure and cardiac output are compromised postsepsis, but subsequently stabilize over the 24-h recording period. In contrast, mesenteric blood flow continuously declines in a time-dependent manner and in parallel with the development of metabolic acidosis and organ dysfunction. Importantly, these microcirculatory perturbations are reversed by fluid resuscitation, a mainstay intervention associated with improved outcome in patients. These data suggest that global hemodynamics are maintained at the expense of the microcirculation and are, therefore, not sufficiently predictive of outcome. We demonstrate that microcirculatory blood flow is a more sensitive biomarker of sepsis syndrome progression and believe that incorporation of this biomarker into preclinical models will facilitate sophisticated proof-of-concept studies for novel sepsis interventions, providing more robust data on which to base future clinical trials.

sepsis is an overwhelming systemic inflammatory response to infection that can progress to severe sepsis and, ultimately, septic shock, characterized by cardiovascular dysfunction, refractory hypotension, and insufficient organ perfusion. Despite decades of research, sepsis continues to be a significant clinical problem, accounting for an estimated 215,000 deaths per annum in the USA alone (2).

Historically, the treatment of patients with severe sepsis in critical care units has focused on achieving a target blood pressure and cardiac output (CO) and normalizing global circulatory parameters. In recent years, it has become increasingly clear that systemic stabilization does not necessarily prevent the onset of organ failure and may actually occur at the expense of microcirculatory perfusion (43, 45). Indeed, persistently diminished microcirculatory flow in patients with restored blood pressure and cardiac function is associated with organ failure and poor outcome (43). While clinical studies have demonstrated a strong association between microcirculatory perfusion and outcome (15), and microvascular monitoring is increasingly used as a research tool in the clinic (14), preclinical models rarely assess this parameter. This may have contributed to the subsequent failure in clinical trials of numerous promising preclinical leads.

Sepsis and septic shock are commonly modeled in rodents by systemic administration of a bacterial endotoxin, or through puncture of the cecum to induce polymicrobial peritonitis (40). These models produce pathophysiological alterations similar to those encountered in patients (a profound inflammatory response with disrupted thermoregulation and cardiovascular function). Nonetheless, they are widely criticized for their limitations and propensity to generate promising therapies that have later failed in clinical trials (38). This poor clinical translatability may, in part, arise from inadequacies in the prognostic value of measured end points. Indeed while systemic hemodynamics are routinely monitored in preclinical models of sepsis and septic shock, these parameters do not sufficiently predict the onset of organ failure (1, 52), the ultimate cause of death in patients (15).

Shunting of blood flow away from compliant tissues (those supplied by the splanchnic circulation, for example) to maintain perfusion of the brain and heart is thought to occur in sepsis (22, 36). While initially adaptive, prolonged regional ischemia can become maladaptive, since poor intestinal perfusion, known clinically as the “motor of organ failure” (7), can actually exacerbate systemic inflammation by facilitating breakdown of intestinal barriers (44). Correspondingly, gastric tonometry and sublingual blood flow have previously been used as clinical tools to ascertain patient prognosis and evaluate the response to therapeutic interventions (37, 48).

Given that splanchnic perfusion can be used clinically as an indicator of syndrome progression, it would seem prudent to assess this parameter during preclinical experimentation. Furthermore, a greater understanding of microcirculatory disturbances in sepsis may help to guide therapeutic intervention. Indeed, it is known that patients become resistant to catecholamine vasopressor treatment, and prolonged use of such agents may worsen outcome (17). Similarly, many vasoactive agents have divergent effects in macrocirculatory vs. microcirculatory beds (18), and hence empirical measures of both, taken in parallel as the sepsis syndrome progresses, seem essential both for preclinical studies, to validate new drug targets, and for the clinical management of sepsis patients.

We have developed and validated a novel approach to assessing microcirculatory perfusion in septic mice and have used this in conjunction with traditional measures of global circulatory hemodynamics, locomotor activity, thermoregulation, cardiac function, and blood biochemistry. We present data from two widely used models of sepsis where we compare and describe, in absolute terms, the observed pathophysiological changes at all levels of the cardiovascular system in a single mouse. Our multiparameter monitoring system facilitates paired analysis, where each animal acts as its own control, maximizing the data generated, while minimizing stress-induced artifacts. This has allowed us to investigate the prognostic value of different cardiovascular parameters. We demonstrate that, while macrocirculatory hemodynamics stabilize during the course of the sepsis syndrome development, it is microcirculatory perfusion that more closely correlates with markers of end-organ damage and metabolic acidosis.

## METHODS

All animal experiments were conducted under a UK Home Office license, following local ethics committee approval and in accordance with the Home Office Animal (Scientific Procedures) Act, 1986. Experiments were designed and conducted in a blinded manner and in accord with the ARRIVE (Animal Research: Reporting of In Vivo Experiments) guidelines (30).

### 

#### Murine models of septic shock.

Male C57BL/6 mice were bred in house and given access to food and water ad libitum. Sepsis was induced under brief anesthesia (2% isoflurane; by air pump). Endotoxemia was induced by intravenous injection into the tail vein of lipopolysaccharide (LPS; 12.5 mg/kg) from *Salmonella* typhimurium (Sigma, L7261). Polymicrobial peritonitis was induced by cecal ligation and puncture (CLP), as described previously (40). Briefly, the cecum was externalized through a midline laparotomy, then ligated and punctured through and through with a 19-gauge needle. Slight pressure was applied to extrude a single droplet of fecal matter from each of the two puncture sites. In control sham-operated animals, laparotomy and exteriorization were performed without ligation and puncture. Incisions were closed with 5.0 Vicryl sutures, and saline resuscitation (0.9%; 40 ml/kg sc) was given. Buprenorphine hydrochloride (15 μg/kg im; Vetergesic; Alstoe Animal Health, UK) was administered to provide postoperative analgesia.

#### Implantation of radiotelemeters and hemodynamic measurements.

PA-C10 radiotelemeter devices (Data Sciences International; DSI) were implanted in 10-wk-old mice under isoflurane anesthesia (2% by air pump). Mice were kept on a homeothermic heating blanket (Harvard Instruments) with eye protection (Viscotears). The surgical field was sterilized with chlorhexidine, and buprenorphine hydrochloride (15 μg/kg im) was administered to provide postoperative analgesia. Telemeter catheters were implanted into the left carotid artery of naive mice and advanced toward the aortic arch. The body of the transmitter was placed in a subcutaneous pocket in the left flank, equidistant between the fore- and hindlimb. The incision was closed with 5.0 Vicryl sutures. Saline resuscitation (0.9%; 40 ml/kg sc) was administered, and animals were placed in a recovery cabinet at 28°C for 4 h. Animals were allowed to recover at room temperature for 10 days before recording of baseline parameters over 48 h. Sepsis was then induced as described above, and recordings were taken over a further 24 h. Animals exhibiting a dampening of the hemodynamic profile were excluded from analysis. Radiotelemetry devices allow continuous remote recording of blood pressure waveforms and cage activity. Data were acquired continuously at 25 Hz using standard acquisition software (DSI).

#### Evaluation of cardiac performance by echocardiography.

Left ventricular (LV) function and chamber dimension were determined in vivo by echocardiography under isoflurane anesthesia. Mice were placed in a supine position on a homeothermically controlled table, and limb leads were attached for electrocardiogram gating. Images were acquired in the left lateral decubitus position with a 30-MHz linear probe (Visualsonic Vevo 770, 30-MHz linear signal transducer). Two-dimensional images in parasternal long- and short-axis projections were recorded with guided M-mode recordings at the midventricular level in both views. Interventricular, septal, and LV posterior wall dimensions were taken in diastole and systole, in addition to LV internal dimensions.

#### Evaluation of blood flow by laser speckle contrast imaging.

Blood flow was recorded in the mesenteric vasculature and the ear, using a moorFLPI full-field laser perfusion imaging system and review software (Moor Instruments, Devon, UK), either in control animals, or at various time points after the induction of sepsis (6 or 24 h post-LPS or post-CLP).

Mice were anesthetized under isoflurane (2% by air pump), and core temperature was recorded and controlled by a rectal probe coupled to a homeothermic heating mat. For mesenteric blood flow measurements, mice were laid on their side, hair was removed from the abdomen by electrical shaver, and a small midline incision was made. A portion of the small intestine was gently exteriorized onto a parafilm-coated heating mat and was pinned out through the gut wall to expose the mesenteric vasculature (see Fig. 2*A*). Care was taken not to stretch or puncture blood vessels, and, if excessive bleeding was observed, mice were terminated immediately and excluded from analysis (∼7%). The exposed vascular bed was kept moist with saline (0.5 ml aerosolized) prewarmed to 37°C. For ear measurements, mice were placed in the prone position. The laser was positioned ∼25 cm above the region of interest, and zoom and focus were adjusted appropriately to acquire high-resolution images of the ear or mesentery (see Fig. 2*A*). No further investigator intervention or microdissection was necessary.

The following acquisition modes and settings were used: high-resolution capture (25 frames, 1 s/frame); exposure 20 ms; automatic gain; flux palette set at 0–5,000 for mesentery, and 0–1,000 for ear; background threshold 60 flux units. Flux over time was analyzed offline by moorFLPI Review software (V3; Moor Instruments, Devon, UK) and branches of the mesenteric vascular tree were designated as first-, second-, or third-order vessels. Regions of interest in which flux over time was measured were defined in each visible vessel, and a mean value was obtained for each level of mesenteric branching in each vascular bed. All assessment and analysis were performed in a blinded manner.

This system is based on the random speckle patterns produced when tissue is illuminated by laser light. In regions of high blood flow, the speckle pattern generated by moving red blood cells becomes blurred, reducing contrast in that region. Correspondingly, high-contrast speckle patterns are associated with low flow. Automated processing of the contrast images generates real-time, high-resolution, color-coded flux images, which correlate with blood flow in each region (6).

#### Blood biochemistry.

Following mesenteric blood flow recording, a venous blood sample was drawn from the inferior vena cava. Blood biochemistry was assessed immediately from 100-μl venous blood using a hand-held iSTAT point-of-care analyzer (Abbott Laboratories), with CG8+ cartridges (Abbott Laboratories). The remaining blood was centrifuged for isolation of the plasma fraction and snap-frozen for future analysis. Animals were terminated by cervical dislocation, and organs were harvested and snap-frozen for future biochemical analysis.

#### Assessment of clinical relevance.

In a separate series of experiments, the impact of two mainstay clinical interventions on microcirculatory blood flow was assessed. Following CLP surgery, animals were randomized to receive either no intervention, or Hartmann's solution containing 5% dextrose (40 ml/kg sc) at 0, 3, 6, and 18 h postsepsis, based on a regimen known to attenuate mortality in septic mice (51). This fluid, similar to lactated Ringer's solution, is a crystalloid compound sodium lactate solution that is closely isotonic with blood and is frequently used in the intensive care unit. In LPS-treated mice, an intravenous catheter was introduced into the left jugular vein under isoflurane anesthesia (2% by air pump) immediately before blood flow measurement. After a 5-min baseline recording, a bolus dose of saline (100 μl over 10 s) was administered intravenously, followed 5 min later by a bolus dose of norepinephrine bitartrate (30 μg/kg in saline; 100 μl over 10 s).

The impact of a topical vasodilator was also assessed in LPS-treated mice, to determine reversibility of mesenteric flow perturbations. Sodium nitroprusside (10 μM in saline) was administered to the mesentery as an aerosolized spray (∼200 μl from a distance of 10 cm) after 5-min baseline recording.

All administered solutions were prewarmed to 37°C before administration.

Microcirculatory blood flow and blood biochemistry were assessed as described above. All studies were conducted in a blinded manner, and investigators were unblinded to treatment group following data analysis.

#### Statistical analysis.

Data are represented as means ± SE from *n* number of mice, unless otherwise indicated. Data were assessed for normality of distribution, and statistical comparisons of means were performed using a Student's *t*-test, or an ANOVA, as appropriate. Results were considered statistically significant when *P* < 0.05. Data were analyzed using GraphPad Prism 5.0 or SPSS v.21 software.

## RESULTS

### 

#### Assessment of systemic hemodynamics by radiotelemetry.

**Fig. 1.**
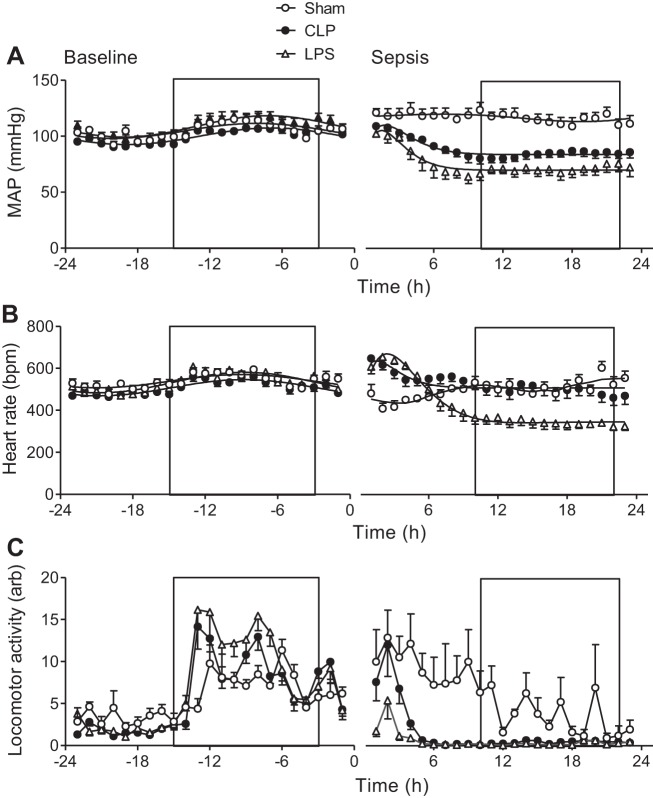
Assessment of systemic hemodynamics and locomotor activity in the same mice under naive and septic conditions. Mean arterial pressure (MAP; *A*), heart rate (*B*), and locomotor activity (*C*), before and after induction of sepsis [at *time 0*: lipopolysaccharide (LPS), 12.5 mg/kg, or cecal ligation and puncture (CLP)] or sham procedures are shown. Boxed regions denote periods of darkness. Data were acquired by radiotelemetry in conscious, ambulatory male C57BL/6 mice and are presented as means ± SE; *n* = 6–11. bpm, Beats/min.

Before the induction of sepsis, mean arterial pressure (MAP) and heart rate (HR) exhibited normal diurnal variation, within the expected physiological range for mice (12), and data were fitted to a sine-wave function describing diurnal variation (Fig. 1, *A* and *B*, *left*) (5). Following the induction of sepsis, the diurnal variation was lost, and data were consequently fitted to a sine wave with exponential function (Fig. 1, *A* and *B*, *right*). Diurnal variation in locomotor activity was also lost following the induction of sepsis (Fig. 1*C*).

A paired comparison revealed a significant difference in MAP between the naive and septic states for both CLP- and LPS-treated mice (Table 1). A separate cohort of sham-operated mice was included to determine the impact of laparotomy on diurnal hemodynamics and activity and to be used as a surgical control for CLP-treated animals. MAP following CLP was significantly reduced relative to sham-operated controls, while no significant difference in MAP was detected between paired naive and sham states (Table 1). The same pattern of statistically significant change was observed for both systolic and diastolic blood pressures in septic mice, but not in sham-operated animals (data not shown).

**Table 1.** Statistical comparison of hemodynamic data obtained by radiotelemetry

	Mean Arterial Pressure, mmHg			Heart Rate, beats/min		
	Mean (CI)	df	*P* value	Mean (CI)	df	*P* value
Difference naive-CLP	−11 (−5 to −17)	10	0.003*	+21 (−79 to +37)	10	0.4425
Difference naive-LPS	−37 (−25 to −49)	9	<0.0001†	−112 (−64 to −161)	9	0.007*
Difference naive-sham	+11 (−27 to +4)	4	0.1122	−34 (23 to −45)	4	0.0009†
Difference sham-CLP	+28 (+16 to +41)	24	0.0002†	−30 (−79 to +20)	24	0.2316

Values are the difference in parameter estimates for the mean [with 95% confidence interval (CI) in parentheses], with degrees of freedom (df) over a 24-h period (either before or following sepsis or sham surgery); *n* = 6–11 mice. Values correspond to graphical data depicted in Fig. 1. CLP, cecal ligation and puncture; LPS, lipopolysaccharide.

* *P* < 0.01 and

† *P* < 0.001, paired or unpaired two-tailed Student *t*-test.

While LPS-treated mice became steadily more hypotensive postsepsis, mice subjected to CLP showed a more gradual decrease in MAP that was less marked than in the LPS model (Fig. 1*A*). This may be explained by the accelerated nature of the LPS model, which bypasses bacterial processing and opsonization steps and is consistent with previous hemodynamic assessments in murine endotoxemia (39). In both models, however, MAP was found to stabilize after 9 h postsepsis, a phenomenon that was not attributable to nocturnal increases in locomotor activity (as this was dramatically and persistently reduced as of 4 h postsepsis; Fig. 1*C*) and was, therefore, likely the result of compensatory cardiovascular changes.

Consistent with a decline in blood pressure, a transient period of tachycardia was observed in both models of sepsis (Fig. 1*B*). This reflex was subsequently lost in LPS-treated mice, which went on to develop a significant reduction in HR relative to the paired naive state (Table 1). In contrast, CLP-treated mice did not exhibit a significantly altered HR, relative to the paired naive state, and no significant difference in HR was detected between sham- and CLP-operated mice (Table 1). Sham surgery caused a transient decline in HR, consistent with the stress-induced hypertension (Fig. 1*A*), suggesting an intact baroreceptor reflex in these mice (Fig. 1*B*).

#### Assessment of cardiac function by echocardiography.

Cardiac function was measured in anesthetized telemetered mice before and after the induction of sepsis. In both models of sepsis, CO and stroke volume were found to decline initially, indicating impaired cardiac function as of 6 h, consistent with previous reports (27, 29, 51) (Table 2). Neither the presence of a telemetry probe during echocardiographic recording, nor the induction of anesthesia and echocardiographic monitoring in telemetered mice was found to alter the hemodynamic profile relative to uninstrumented mice or previous reports (27, 29, 35, 46, 50, 51), suggesting that both techniques can be used in concert without mutual interference.

**Table 2.** Cardiac function before and after induction of sepsis

	Sham	6-h CLP	24-h CLP	Naive	6-h LPS	24-h LPS
Cardiac output, ml/min	27.2 ± 1.5	19.7 ± 1.5†	18.3 ± 1.6‡	32.9 ± 1.4	22.0 ± 1.9‡	22.0 ± 1.1‡
Stroke volume, μl	49.6 ± 2.5	38.8 ± 3.7*	26.7 ± 3.9*	60.0 ± 2.5	44.9 ± 4.9*	43.7 ± 1.3*
End-diastolic left ventricular volume, μl	78.8 ± 5.2	59.1 ± 5.8	48.8 ± 6.2†	94.3 ± 3.6	116.1 ± 9.1	77.7 ± 9.4
Ejection fraction, %	63.8 ± 2.4	66.2 ± 2.7	77.4 ± 3.3*	63.4 ± 2.4	39.5 ± 2.0‡	57.1 ± 6.4
Fractional shortening, %	34.6 ± 1.8	36.2 ± 2.1	46.4 ± 3.5†	34.5 ± 1.8	19.3 ± 1.1†	30.6 ± 4.3
Aortic flow, mm/s	1438 ± 82	1337 ± 98	1152 ± 63	1519 ± 56	808 ± 36‡	1136 ± 97*

Values are means ± SE, *n* = 6–8 mice. Cardiac function was recorded by echocardiography (Vevo 770) in homeothermically heated (to 36°C), unconscious (1.8% isofluorane) naive and sham-operated animals, and 6 and 24 h post-LPS and post-CLP.

* *P* < 0.05,

† *P* < 0.01,

‡ *P* < 0.001 relative to sham/naive, one-way ANOVA + Bonferroni post hoc test.

#### Characterization of mesenteric perfusion by full-field laser speckle contrast imaging.

**Fig. 2.**
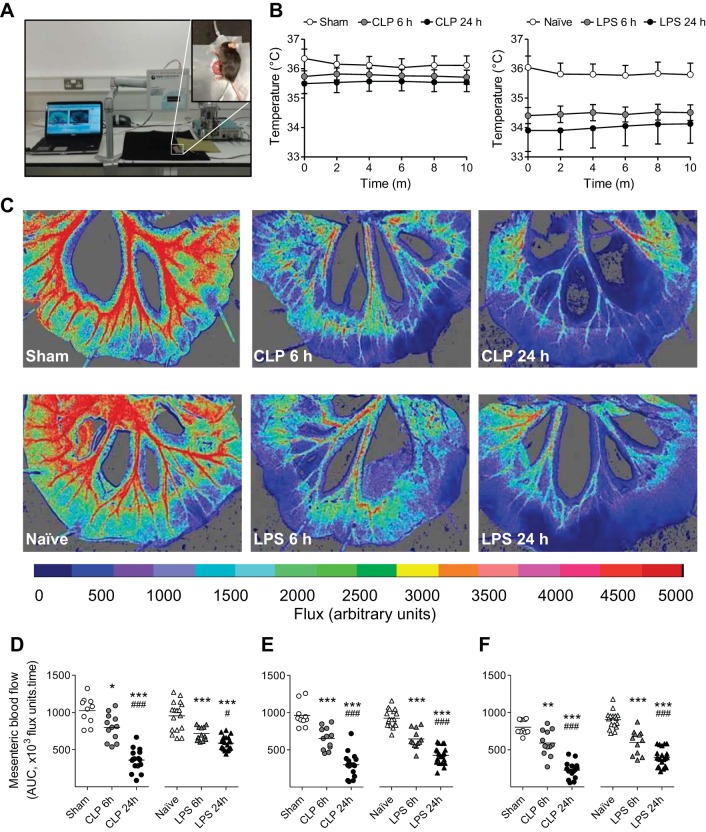
Assessment of mesenteric blood flow in healthy and septic mice. *A*: experimental setup involving exteriorization of a section of the small intestine through a small abdominal incision and careful pinning through the gut wall to expose the mesenteric vascular bed. Mice were placed on a parafilm-coated homeothermic blanket coupled to a rectal temperature probe. The exposed mesenteric bed was moistened with prewarmed aerosolized saline solution. *B*: core body temperature measured by rectal probe during baseline blood flow recording in naive and sham-operated animals and at 6 and 24 h post-LPS and -CLP (means ± SE; *n* = 10–21). *C*: representative flux images taken during baseline recording. Cold colors indicate low flow; warmer colors higher flow. Mesenteric blood flow is expressed as total area under the curve (AUC; ×10^3^ flux units/time) over 5-min baseline recording in first (*D*), second (*E*), and third-order vessels (*F*), in naive and sham-operated animals and 6 and 24 h post-LPS and -CLP. AUC for each animal is represented as an individual symbol, with mean for each group denoted by a horizontal line. *P < 0.05, **P < 0.01, ***P < 0.001 relative to naive/sham, and #*P* < 0.05, ###*P* < 0.001 relative to 6 h (one-way ANOVA + Bonferroni post hoc test).

Assessment of mesenteric or subcutaneous ear blood flow was performed in situ under isoflurane anesthesia and using a homeothermically controlled heating mat coupled to a rectal temperature probe (Fig. 2*A*). Both core temperature (Fig. 2*B*) and mesenteric blood flow remained stable throughout the recording period, and for up to 60 min (data not shown). The presence of a telemetry probe in the flank of the mouse had no bearing on the flow values recorded. LPS-treated mice exhibited a hypothermic phenotype at 6 h that was partially reversed by 24 h. CLP-treated mice became gradually, but less severely, hypothermic over 24 h. These observations are consistent with previous reports (8, 10, 42).

In contrast to microcirculatory hemodynamics, which stabilized between 9 and 24 h postsepsis (Fig. 1 and Table 2), mesenteric blood flow continued to decline in a time-dependent manner, in both models of sepsis and in all vessel orders (Fig. 2, *D*–*F*).

#### Systemic venous blood biochemistry.

Consistent with impaired microcirculatory perfusion, blood pH was significantly reduced in a time-dependent manner in both models of sepsis. Similarly, creatinine and urea were markedly elevated at 24 h post-LPS and -CLP compared with the 6-h time point, indicating end organ dysfunction despite systemic blood pressure and CO stabilization. The increase in creatinine and urea levels observed here is of a similar magnitude to those reported previously both for LPS (9, 10) and CLP (23, 49). Mice also became severely hypoglycemic over the 24-h time period, indicative of metabolic disturbance similar to that observed in humans subjected to experimental endotoxemia (48). Venous oxygen saturation (So_2_) did not change significantly over the course of sepsis in either model, however. These data and all other blood biochemistry are summarized in Table 3.

**Table 3.** Blood biochemistry in healthy and septic mice

	Sham	6-h CLP	24-h CLP	Naive	6-h LPS	24-h LPS
Creatinine, μmol/l	18 ± 0.0	18 ± 0.0	36.5 ± 29.3*	18 ± 0.0	18 ± 0.0	40.18 ± 14.8*
Urea, mmol/l	4.79 ± 0.29	6.08 ± 0.67	17.03 ± 4.26	4.57 ± 0.37	8.10 ± 1.45	36.47 ± 2.89‡§
pH	7.29 ± 0.01	7.23 ± 0.02	7.15 ± 0.03†	7.29 ± 0.03	7.25 ± 0.02	7.13 ± 0.03†
Base excess, mmol/l	−11.87 ± 0.80	−17.00 ± 0.97	−15.21 ± 1.19	−11.0 ± 0.78	−12.60 ± 1.72	−17.19 ± 1.22‡
HCO_3_^−^, mmol/l	15.6 ± 1.2	10.9 ± 2.0*	13.9 ± 2.0	17.8 ± 2.0	12.8 ± 4.1*	12.8 ± 4.7†
So_2_, %	87.8 ± 4.2	83.7 ± 6.7	80.8 ± 12.8	89.7 ± 1.5	88 ± 9.2	78.5 ± 6.9
Glucose, mmol/l	12.6 ± 3.6	4.8 ± 1.0‡	3.0 ± 1.3‡	13.2 ± 3.5	3.2 ± 1.4‡	2.6 ± 1.5‡
Lactate, mmol/l	3.8 ± 0.7	2.6 ± 0.4	4.6 ± 1.2	3.9 ± 0.0	1.6 ± 0.6	3.2 ± 1.0
Na^+^, mmol/l	147.2 ± 1.8	154.0 ± 2.6†	149.3 ± 2.7	147.6 ± 2.4	152.5 ± 2.7*	150.9 ± 4.8
K^+^, mmol/l	4.8 ± 1.1	3.3 ± 0.6	4.9 ± 1.9	4.2 ± 1.6	3.3 ± 0.4	4.5 ± 1.7
Cl^−^, mmol/l	121.8 ± 3.2	128.0 ± 2.2	126.3 ± 4.9	121.7 ± 3.9	128.2 ± 1.5*	129.1 ± 5.7†
Hemoglobin, g/dl	11.3 ± 1.1	9.8 ± 0.7	10.4 ± 1.8	11.4 ± 2.2	8.6 ± 1.6	10.6 ± 2.3
Hematocrit, %PCV	32.6 ± 1.1	26.25 ± 2.6	30.57 ± 1.4	33.43 ± 2.0	26.0 ± 1.9	30.25 ± 2.1

Values are means ± SE; *n* = 3-16 mice.

HCO_3_^−^, bicarbonate; So_2_, venous oxygen saturation; PCV, packed cell volume.

* *P* < 0.05,

† *P* < 0.01, and

‡ *P* < 0.001 relative to sham/naive; and

§ *P* < 0.001 relative to 6-h time point, one-way ANOVA and Bonferroni post hoc test.

* <18 μmol/l cut-off value on iSTAT device.

#### Effect of fluid resuscitation on microvascular and metabolic function.

Given the continued deterioration of mesenteric blood flow over the 24-h period, compared with the apparent preservation of macrocirculatory hemodynamics and global So_2_, we aimed to ascertain whether our technique for quantifying microcirculatory blood flow was sensitive to fluid resuscitation, a mainstay clinical intervention known to improve microcirculatory perfusion, organ function, and survival in patients (41) and septic mice (21).

**Fig. 3.**
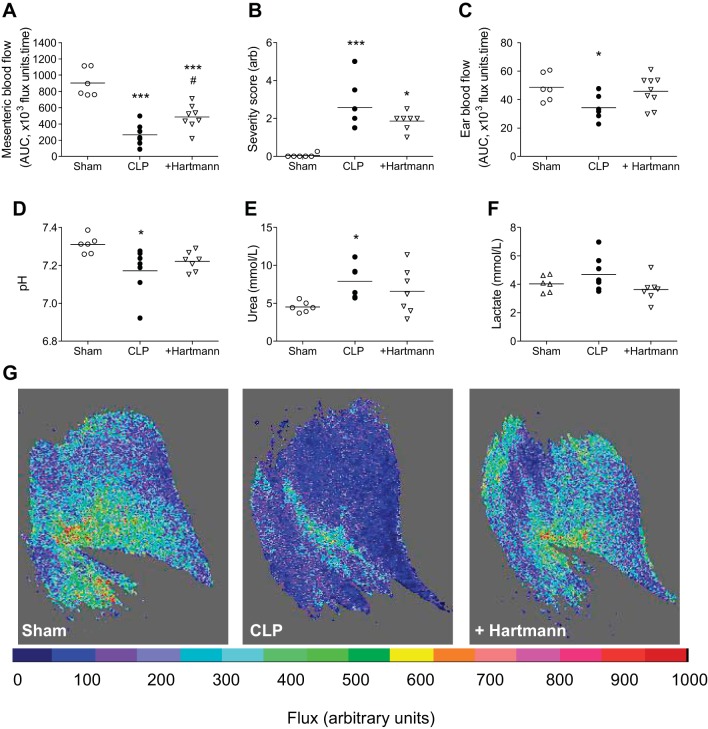
Impact of Hartmann's fluid resuscitation on sepsis parameters. Hartmann's resuscitation (40 mg/kg sc; +Hartmann) was administered immediately after CLP and at 3, 6, and 18 h thereafter. Control CLP-treated animals were given a bolus injection immediately postsurgery, but received sham injections at later corresponding time points. *A*: blood flow in first-order mesenteric vessels expressed as AUC (×10^3^ flux units/time) over 5-min baseline recording. *B*: severity score (scale 0–5, arbitrary units) assigned in a blinded fashion based on characteristics such as voluntary mobility, gait, aversion to touch, facial expression, and piloerection. *C*: subcutaneous ear blood flow expressed as AUC over 3-min baseline recording. Blood pH (*D*), blood urea (*E*), and blood lactate (*F*) were measured from venous blood samples by iSTAT point-of-care analyzer. Measurements for each animal are represented as an individual symbols (*n* = 6–9) with mean for each group denoted by a horizontal line. *P < 0.05, ***P < 0.001, relative to sham, and #*P* < 0.05, relative to CLP (one-way ANOVA + Bonferroni post hoc test). *G*: representative flux images of subcutaneous ear blood flow. Cold colors represent areas of low blood flow; warmer colors, areas of high blood flow.

This intervention modestly, but significantly, improved perfusion in first-order mesenteric vessels (Fig. 3*A*), and a similar pattern of improvement was observed in second- and third-order vessels [mean area under the curve (×10^3^ flux units/time): 221 ± 52 vs. 407 ± 78 in second-order vessels, nonsignificant; 141 ± 334 vs. 343 ± 44 in third-order vessels, *P* < 0.05]. Volume-resuscitated mice also exhibited modest improvements in hypothermia (data not shown), arbitrary severity score (based on posture, mobility, aversion to touch, and piloerection, assigned in a blinded fashion; Fig. 3*B*), metabolic acidosis (Fig. 3*D*), urea (Fig. 3*E*), and lactate production (Fig. 3*F*). Interestingly, measurement of perfusion by the same method in a different microvascular bed, the ear, revealed a significant decrease in blood flow in CLP-treated mice that was also reversed by fluid resuscitation (Fig. 3, *C* and *G*).

**Fig. 4.**
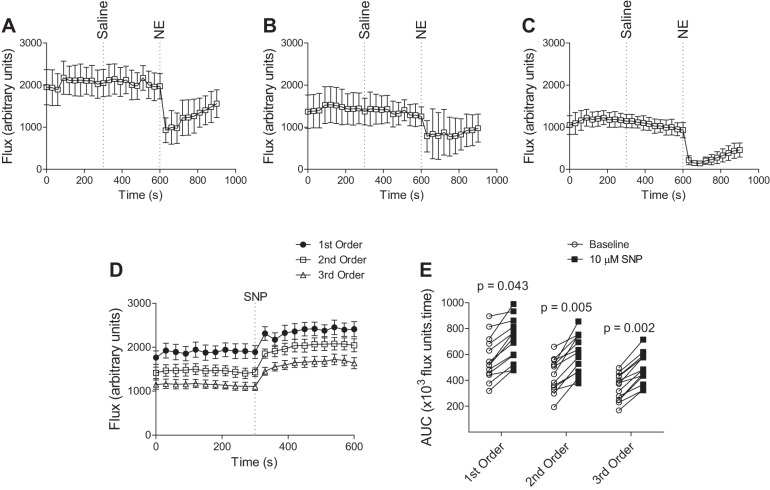
Impact of vasoactive agents on mesenteric blood flow in LPS-treated mice. *A*–*C*: effect of systemic norepinephrine (NE) in first-order (*A*), second-order (*B*), and third-order (*C*) branches of the mesenteric vascular tree. Following a 5-min baseline recording, saline (100 μl) was administered systemically, via an intravenous catheter in the left jugular vein, followed by a bolus injection of NE (30 μg/kg in 100 μl saline) 5 min later. Values are means ± SE; *n* = 5. *D* and *E*: effect of topical vasodilator sodium nitroprusside (SNP; 10 μM in saline) on mesenteric blood flow in first-, second-, and third-order vessels. *D*: flux recorded over 5-min baseline and following topical administration of SNP (2 pumps of aerosolized spray). *E*: AUC (×10^3^ flux units/time) for each mouse over 5-min baseline recording and 5-min response to SNP. Measurements for each animal are denoted by individual symbols (*n* = 12), and comparisons of baseline AUC with AUC post-SNP were assessed by Student's unpaired two-tailed *t*-test.

Intravenous bolus administration of norepinephrine, known to raise MAP in endotoxic mice (39), did not improve mesenteric perfusion in LPS-treated mice. On the contrary, it caused a marked and relatively sustained decrease in blood flow (Fig. 4, *A*–*C*). These data support the notion that improvement in global hemodynamic parameters does not necessarily result in improved regional perfusion, and that the pharmacological activity of vasopressors, such as norepinephrine (α_1_-adrenoceptor-mediated smooth muscle constriction), may override the physiological effect of increasing global oxygen delivery. Topical administration of sodium nitroprusside, a vasodilator, on the other hand, was found to reverse LPS-induced microvascular impairment, in all order branches of the mesenteric vascular tree (Fig. 4, *D* and *E*). These data suggest that active vasoconstriction accounts, at least in part, for the reduced mesenteric perfusion observed in sepsis and are consistent with the effects of topical vasodilators applied to the sublingual microvasculature in septic patients (13).

## DISCUSSION

The key findings from this study are as follows:

*1*) Laser speckle contrast imaging can be used in concert with other standard hemodynamic techniques to robustly assess microcirculatory perfusion in septic mice.

*2*) Microcirculatory flow, as quantified in the mesenteric circulation, more closely correlates with end-organ perfusion and metabolic disturbances in both sepsis models.

*3*) Stabilization of macrocirculatory hemodynamics (MAP and CO) is likely to occur at the expense of microcirculatory perfusion and is, therefore, insufficiently predictive of outcome. Similarly, strategies aimed at elevating MAP pharmacologically may adversely affect microcirculatory perfusion.

*4*) Aggressive fluid resuscitation measurably improves microcirculatory perfusion and end-organ function, consistent with clinical observations.

A strong research drive and extensive media coverage have served to bring sepsis into public prominence in recent years. A significant factor in the lack of effective treatments for septic shock, in addition to the limitations of related clinical trials (28), is the problem of research translatability; numerous interventions that have proved beneficial in rodent models have failed to translate into improved outcome in humans (38). Patient and pathogen heterogeneity, and the confounding effects of clinical intervention, complicate the process of developing a relevant model in which to investigate disease mechanisms. Preclinical focus on end points with low prognostic value, however, may also have contributed significantly to this issue. While research has tended to center on global hemodynamics and crude mortality end points, models that focus on the mechanism of death, specifically perfusion failure, may be more clinically relevant.

**Fig. 5.**
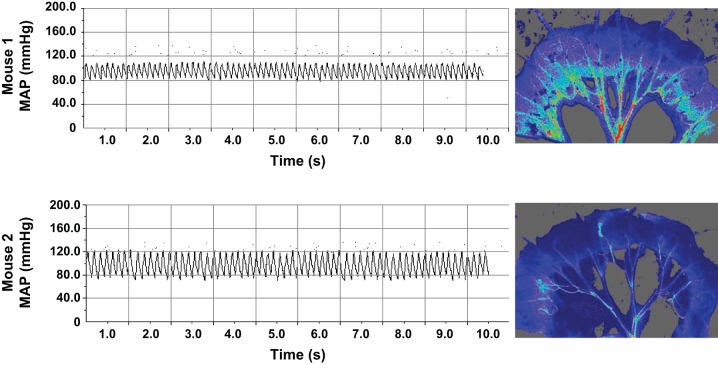
Representative blood pressure traces and corresponding mesenteric blood flow images from two LPS-treated mice. Blood pressure traces were collected immediately before blood flow recording at 24 h post-LPS. Whereas *mouse 1* exhibited compromised MAP and pulse pressure, mesenteric perfusion was less severely disrupted relative to *mouse 2*, which exhibited a profound decrease in mesenteric perfusion, despite normal hemodynamics. This demonstrates that MAP alone is insufficiently predictive of syndrome progression.

In this study, it is especially noteworthy that, in both models, sepsis-induced hypotension and alterations in CO and HR stabilized after 9 h, with values at 24 h equivalent to those at 6 h postsepsis, despite a continued decline in mesenteric perfusion. This suggests that systemic stabilization may be preserved at the expense of the microcirculation, as discussed previously (45), and as demonstrated clearly in Fig. 5. Furthermore, while MAP and HR data suggested that LPS-treated mice displayed marked cardiovascular dysfunction, the equivalent changes in CLP-treated mice were relatively modest. In contrast, microcirculatory perfusion in CLP-treated mice was markedly impaired and continued to decline over the experimental time course, correlating closely with clinically relevant markers of outcome (i.e., metabolic acidosis and organ dysfunction). Moreover, administration of norepinephrine, known to raise arterial pressure and used clinically to restore hemodynamics, actually diminished mesenteric perfusion, suggesting, first, that prolonged vasopressor use may accelerate organ failure, and, second, that treatment strategies targeted at global hemodynamics may have limited efficacy, as demonstrated previously (19). Furthermore, these data emphasize the importance of titrating vasopressor doses carefully, to limit the potential for detrimental effects on organ perfusion.

Taken together, these observations highlight the importance of measuring loco-regional perfusion directly, rather than using global surrogates. Numerous studies have demonstrated that restoration of global oxygen delivery does not necessarily enhance regional perfusion (4, 16, 19, 26, 33). A potentially large contributing factor in this “oxygen extraction deficit” is the active constriction of compliant vascular beds (including the gut) to elevate peripheral vascular resistance and arterial pressure. This may account for the measurements of normal mixed-venous So_2_ levels, despite severe local tissue dysoxia, as observed both in this study and in septic patients (24). Consistent with this hypothesis, we have demonstrated a significant increase in blood flow through regional administration of a vasodilatory agent, indicating that mesenteric flow disruption in sepsis comprises, at least in part, an active vasoconstrictive component.

While acute arteriovenous shunting may be essential to preserve global circulation and perfusion of heart and brain tissue in acute hypovolemia, prolonged microcirculatory shutdown, most notably in the splanchnic region, inevitably leads to hypoxic tissue injury and the development of organ failure (45, 47). Clinically described as “the motor of multiple organ failure” (7), the gut represents an important microcirculatory bed in septic shock, where impaired flow is known to correlate strongly with poor outcome both in animal models (3) and sepsis patients (45, 47). This likely reflects the fact that impaired perfusion of the intestine, and associated hyperpermeability, facilitates the leakage of microorganisms and endotoxin from the gut lumen into the lymphatic and cardiovascular circulation, exacerbating the inflammatory state (44).

Correspondingly, our data emphasize that stabilization of macrocirculatory parameters may not indicate halting of syndrome progression and, therefore, should not be used as a prognostic marker in isolation. This conclusion is supported by clinical evidence: numerous trials of interventions that improve hemodynamic performance in animal models (11, 47) and humans have failed to confer a survival benefit in clinical trials (20, 31, 34). We have demonstrated that direct measurement of mesenteric perfusion, known to confer high prognostic value in the clinic (20, 25, 37), is not only feasible in a murine model, it is also more sensitive to syndrome progression.

**Fig. 6.**
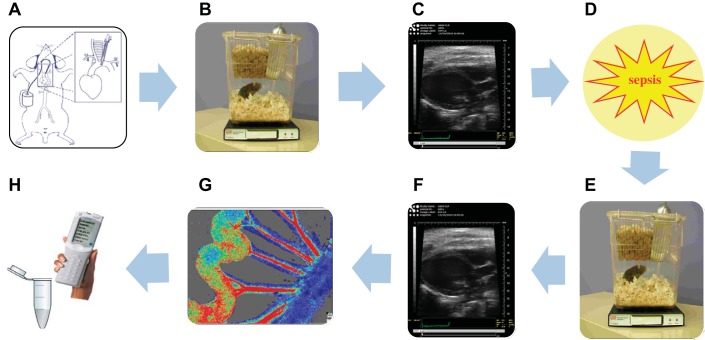
Schematic representation of multiparameter monitoring of sepsis progression in a single mouse. Following implantation of telemetry catheters (*A*), baseline hemodynamics are recorded remotely in unrestrained ambulatory mice (*B*). Basal cardiac function is measured under isoflurane anesthesia by echocardiography (*C*). Sepsis is then induced either by LPS or CLP (*D*), and hemodynamics (*E*) and cardiac function (*F*) are re-recorded in the same mice under pathological conditions. Mice are then terminally anesthetized at a time point of interest, and mesenteric blood flow is measured by laser speckle contrast imaging (*G*). Intravenous or topical drug administration may be used to determine vasoactivity. Mice are then terminated by venupuncture, and blood samples obtained from the inferior vena cava are analyzed by iSTAT point-of-care analyzer (*H*). Further biochemical and histological investigation may then be carried out.

Because murine models facilitate gene modification, they currently represent the only viable option for conducting mechanistic preclinical proof-of-concept research. Therefore, the authors consider it of paramount importance to optimize the systems available to us by limiting confounding influences, and to employ animal models to their full capacity, extracting as much information as possible from a single individual. We have demonstrated that a number of biological measurements can be performed in a single mouse (as shown schematically in Fig. 6) without compromising data quality or reproducibility, and without incurring undue suffering, which can lead to stress-induced artifacts.

This novel application of laser speckle contrast imaging technology enables real-time, in situ visualization of blood flow across the entire mesenteric bed, with no dissection of perivascular tissue and minimal mechanical disruption to the vessels. This represents a significant advantage over traditional laser Doppler techniques that are usually limited to measurements in single vessels, often requiring extensive microdissection and probe-tissue contact, with significantly lower processing speeds. Additionally, this technique does not require the use of fluorescent dyes necessary for intravital microscopy. Furthermore, it allows quantification of flow within specified regions of interest, both at baseline and following topical or intravenous administration of a pharmacological agent. As such, the model represents a practical substitute to the side-stream dark-field imaging technique that is used clinically, while circumventing the need for high-level operating expertise, as well as interoperator variability and probe-tissue contact artifacts.

Measurement of blood flow in the ear may represent a less invasive method for repeated-measures testing of microcirculatory function over a specific time course, given that it does not require terminal anesthesia, although further validation in this bed is required. The observed improvement in perfusion and organ function in response to fluid resuscitation suggest that this model is clinically relevant and may be adapted to a comparatively hyperdynamic murine model of sepsis (21). Furthermore, the model is not limited to sepsis research, but has wider applications for in situ investigation of thrombosis or in situ vasoactivity (32).

In summary, we demonstrate that microcirculatory perfusion more closely correlates with end-organ damage (the ultimate cause of death in sepsis patients) and that macrocirculatory hemodynamics alone may have limited prognostic value. We believe that quantification of microcirculatory blood flow, as described, provides a more humane end point, while simultaneously improving the clinical translatability of preclinical sepsis studies.

## GRANTS

This work was funded by British Heart Foundation Grants PG/09/073 and S/10/51/28677.

## DISCLOSURES

No conflicts of interest, financial or otherwise, are declared by the author(s).

## AUTHOR CONTRIBUTIONS

Author contributions: C.A.S., A.S., D.F.T., and M.N. conception and design of research; C.A.S., A.S., C.D.W., and O.R. performed experiments; C.A.S., A.S., C.D.W., O.R., and D.S. analyzed data; C.A.S., A.S., C.T., D.F.T., and M.N. interpreted results of experiments; C.A.S. and A.S. prepared figures; C.A.S. drafted manuscript; C.A.S., A.S., C.D.W., O.R., C.T., D.F.T., and M.N. edited and revised manuscript; C.A.S. and M.N. approved final version of manuscript.
